# Embryonic Thermal Manipulation Affects Ventilation, Metabolism, Thermal Control and Central Dopamine in Newly Hatched and Juvenile Chicks

**DOI:** 10.3389/fphys.2021.699142

**Published:** 2021-06-17

**Authors:** Aline C. G. Rocha, Caroline Cristina-Silva, Camila L. Taxini, Kaoma Stephani da Costa Silva, Virgínia T. M. Lima, Marcos Macari, Kênia C. Bícego, Raphael E. Szawka, Luciane H. Gargaglioni

**Affiliations:** ^1^Department of Animal Morphology and Physiology, College of Agricultural and Veterinarian Sciences, São Paulo State University, São Paulo, Brazil; ^2^State University of Minas Gerais – UEMG, Passos, Brazil; ^3^Department of Physiology and Biophysics, Institute of Biological Sciences, Federal University of Minas Gerais – UFMG, Belo Horizonte, Brazil

**Keywords:** chicken, hypercapnia, hypoxia, incubation, temperature, ventilation, monoamines

## Abstract

The first third of incubation is critical for embryonic development, and environmental changes during this phase can affect the physiology and survival of the embryos. We evaluated the effects of low (LT), control (CT), and high (HT) temperatures during the first 5 days of incubation on ventilation ($\dot{V}$_*E*_), body temperature (Tb), oxygen consumption ($\dot{V}$O_2_), respiratory equivalent ($\dot{V}$_*E*_/$\dot{V}$O_2_), and brain monoamines on 3-days-old (3d) and 14-days-old (14d) male and female chickens. The body mass of LT animals of both ages and sexes was higher compared to HT and CT animals (except for 3d males). The heart mass of 14d HT animals was higher than that of CT animals. Thermal manipulation did not affect $\dot{V}$_*E*_, $\dot{V}$O_2_ or $\dot{V}$_*E*_/$\dot{V}$O_2_ of 3d animals in normoxia, except for 3d LT males $\dot{V}$_*E*_, which was lower than CT. Regarding 14d animals, the HT females showed a decrease in $\dot{V}$_*E*_ and $\dot{V}$O_2_ compared to CT and LT groups, while the HT males displayed a lower $\dot{V}$O_2_ compared to CT males, but no changes in $\dot{V}$_*E*_/$\dot{V}$O_2_. Both sexes of 14d HT chickens presented a greater Tb compared to CT animals. Thermal manipulations increased the dopamine turnover in the brainstem of 3d females. No differences were observed in ventilatory and metabolic parameters in the 3d animals of either sexes, and 14d males under 7% CO_2_. The hypercapnic hyperventilation was attenuated in the 14d HT females due to changes in $\dot{V}$O_2_, without alterations in $\dot{V}$_*E*_. The 14d LT males showed a lower $\dot{V}$_*E*_, during hypercapnia, compared to CT, without changes in $\dot{V}$O_2_, resulting in an attenuation in $\dot{V}$_*E*_/$\dot{V}$O_2_. During hypoxia, 3d LT females showed an attenuated hyperventilation, modulated by a higher $\dot{V}$O_2_. In 14d LT and HT females, the increase in $\dot{V}$_*E*_ was greater and the hypometabolic response was attenuated, compared to CT females, which resulted in no change in the $\dot{V}$_*E*_/$\dot{V}$O_2_. In conclusion, thermal manipulations affect hypercapnia-induced hyperventilation more so than hypoxic challenge, and at both ages, females are more affected by thermal manipulation than males.

## Introduction

Incubation is a period of high plasticity, when the environment experienced by the embryo can significantly alter the physiological systems of post-hatch life (Walstra et al., 2010). Many factors, such as temperature, atmospheric gaseous composition, pathogen challenges, and nutrition, if applied when the organism’s phenotype is most vulnerable, can greatly influence the embryonic development, and can affect, positively or negatively, the growth of the animal (Ono et al., 1994; Burggren, 1998; De Smit et al., 2006; Mortola, 2011; Mortola and Toro-Velasquez, 2013; Morita et al., 2016; do Amaral-Silva et al., 2019; Rocha et al., 2020). Thermal manipulation during the sex-determination phase of incubation [embryonic day (E)0 to E5] had a positive effect on chicken hatchability and secondary sexual characteristics of males and females, possibly due to a higher plasma testosterone concentration in both males and females (Piestun et al., 2013b).

In fact, changes in embryonic temperature can accelerate or slow down embryo growth and/or metabolism (Tazawa and Rahn, 1987; Tazawa et al., 1989; Christensen et al., 1999; Lourens et al., 2005; Mortola and Toro-Velasquez, 2013), altering the development of vital organs, such as the heart and lungs (Molenaar et al., 2011). The severity of these effects depends on the intensity and duration of the stimulus and the phase of incubation (Webb, 1987; French, 1997; Dzialowski et al., 2002; Mortola and Labbè, 2005). Interestingly, previous studies demonstrated that temperature reduction during embryogenesis decreases the occurrence of ascites and modifies thermoregulatory mechanisms in chicks, improving the thermal tolerance to low rearing temperatures after hatching (Shinder et al., 2011; Akşit et al., 2013). Thermal alterations during incubation can also affect brain regions involved in thermoregulation, such as the proportion between warm- and cold-sensitive neurons in the hypothalamus of hatchling and juvenile ducks (Tzschentke and Basta, 2002).

Embryos subjected to warmer temperatures presented higher dopamine and noradrenaline levels in the brain, whereas those incubated at cooler temperatures had reduced hormone levels (von Blumröder and Tönhardt, 2002). Monoamines [noradrenaline (NA), adrenaline (AD), dopamine (DA), and serotonin (5-HT)], key neurotransmitters that have important neurotrophic and morphogenetic roles in the maturation of the central nervous system (CNS), appear early during prenatal development (Buznikov et al., 2003; Deneris and Gaspar, 2018) and are implicated in multiple physiological and pathological brain mechanisms (Haydon et al., 1984, 1987; Zhao and Debski, 2005; Kalueff et al., 2007). Indeed, the monoaminergic systems play an important role in the control of breathing under resting conditions in most vertebrates (Gargaglioni et al., 2008). Previous studies from our laboratory have demonstrated that catecholaminergic neurons of the brainstem exert an inhibitory tonic effect on neonate rats and an inhibitory modulation under hypoxia and CO_2_ exposure in males and females (Patrone et al., 2018, 2020). The role of DA in breathing control is related to its actions in both the peripheral chemoreceptors (López-Barneo et al., 2009) and in the brainstem nuclei (Kline et al., 2002). While the inhibitory effects of DA are observed in the carotid body (Gonzalez et al., 1994; Iturriaga et al., 1994; Prabhakar, 1994); both excitatory (Hedner et al., 1982) and inhibitory (Bolme et al., 1977) effects are observed centrally. As to 5-HT, brainstem serotonergic neurons contribute to the respiratory response to CO_2_ in chicken, playing an excitatory role in CO_2_-drive to breathing (Santos et al., 2017). Therefore, it is possible that monoaminergic system is affected by thermal manipulation during incubation and affects breathing control in chickens.

The effects of external influences during chicken embryonic development on chemosensitivity have already been reported previously (Szdzuy and Mortola, 2007, 2008; Ferner and Mortola, 2009). The authors demonstrated that newly hatched chickens exposed to low O_2_ or high CO_2_ levels during incubation displayed a blunted ventilatory chemosensitivity during hypoxia and hypercapnia, suggesting an alteration in peripheral chemoreceptors. In addition, variations in body temperature (Tb) also change the chemosensitivity of avian intrapulmonary chemoreceptors (Barnas et al., 1983) as well as peripheral and central chemoreceptors in different vertebrate species (Branco and Wood, 1993; Branco et al., 1993; Rocha and Branco, 1998; Bícego-Nahas and Branco, 1999; Mortola and Frappell, 2000). Nevertheless, little is known about the phenotypic plasticity of high and low incubation temperatures on chemosensitivity and thermal responses in newly hatched and juvenile broiler chickens. Given the sensitivity of embryogenesis to environmental temperatures, we hypothesized that chicken embryos developing under different thermal environments might exhibit changes in brain monoamines, ventilation, metabolism, and thermal responses to hypoxia and hypercapnia. To this end, in 3-day-old (3d) and 14-day-old (14d) chickens (males and females), incubated under different temperatures (36, 37.5, and 39°C) for 6 h/day during the first 5 days of incubation, we evaluated changes in ventilation, breathing variability, metabolism, and Tb during hypoxia (10% O_2_) and hypercapnia (7% CO_2_).

## Materials and Methods

### Animals

The protocols were performed according to CONCEA (“Conselho Nacional de Controle de Experimentação Animal”; National Council for Animal Care Control) and approved by the local animal care committee (CEUA—Comissão de Ética no Uso de Animais—FCAV-UNESP; Protocol: 011955/18).

Freshly laid fertilized eggs from 45- to 50-week-old hens of the *Gallus gallus domesticus* lineage, Cobb 500^®^, were supplied by a local commercial hatchery, and were selected by weight (65 ± 5 g) and held at ∼18°C until incubation. The eggs were incubated (incubator Premium Ecologica^®^, Belo Horizonte, MG, Brazil) with an average relative humidity of 60% and automatic turning every hour. The eggs were then subjected to one of the three treatments: control temperature (CT; 37.5°C throughout incubation), low temperature (LT; 36°C for 6 h/day on days 0–5), or high temperature (HT; 39°C for 6 h/day on days 0–5). From day 6 on, the eggs from LT and HT treatments were maintained at 37.5°C until hatched. After hatching, animals were separated into males and females, and were housed in a room with a controlled environment (COBB, 2008), 14:10 h light-dark cycle (lights on at 6:00 AM) with free access to water and food.

### Determination of Pulmonary Ventilation

Measurements of pulmonary ventilation ($\dot{V}$_*E*_) were performed using the whole-body plethysmography method (closed system; Mortola and Frappel, 1998; Espinha et al., 2014; Rocha et al., 2020). Freely-moving 3- and 14-days-old (3d and 14d) chickens were kept in a 1.5-L and 5-L chamber, respectively, and the chambers were ventilated with humidified room air, a hypercapnic gas mixture (7% CO_2_) or a hypoxic gas mixture (10% O_2_; Gama Gases, São Bernardo do Campo, Brazil) for 30 min each. The ambient temperature was controlled to maintain the thermal comfort of the animals (∼32°C for 3d, and ∼27°C for 14d) (COBB, 2008). During $\dot{V}$_*E*_measurements, the flow was interrupted, the chamber was sealed for short periods of time (∼2 min), and the pressure oscillations caused by breathing were monitored using a differential pressure transducer (TSD 160A, Biopac Systems, Santa Barbara, CA, United States). The signals were fed into a differential pressure signal conditioner (DA 100C, Biopac Systems, United States), passed through an analog-to-digital converter, and digitized on a microcomputer equipped with data acquisition software (MP100A-CE, Biopac Systems, United States). The volume was calibrated during each experiment by injecting 1 mL of air into the animal chamber. The tidal volume (V_*T*_) of 14d animals was calculated using the following formula from Drorbaugh and Fenn (1955), adapted by Bartlett and Tenney (1970):

$$V_{T}=\frac{P_{T}}{P_{K}}.V_{K}.\frac{\left[T_{b}\left(P_{baro}-P_{chH_{2}O}\right)\right]}{\left[T_{b}\left(P_{baro}-P_{chH_{2}O}\right)\right]-\left[T_{ch}\left(P_{baro}-P_{bH_{2}O}\right)\right]}$$

where P_*T*_ is the pressure deflection associated with tidal volume, P_*K*_ is the pressure deflection associated with the air volume injected for calibration, V_*K*_ is the air volume injected into the animal’s chamber for calibration, T_*ch*_ is the chamber temperature, T_*b*_ is the animal Tb, P_*baro*_ is the barometric pressure, P_*chH2O*_ is the water vapor pressure of the chamber, and P_*bH2O*_ is the water vapor pressure of the animal.$\dot{V}$_*E*_ and V_*T*_ are presented at ambient barometric pressure and T_*b*_, and are saturated with water vapor at this temperature (BTPS). The P_*baro*_, P_*chH2O*_ and P_*bH2O*_ were calculated indirectly using an appropriate table (Dejours, 1981), assuming that chamber was fully saturated. T_*ch*_ was monitored using a thermoprobe (model 8502-10, Cole Parmer, Chicago, IL, United States).

Regarding 3d animals, some alterations were needed to guarantee that the animals were within the comfort ambient temperature, while still being able to obtain accurate V_*T*_ measurements. For this, the newly hatched animal was placed individually inside a chamber made of two separate compartments connected through a tube. The T_*ch*_ of the animal chamber was controlled by a water bath and maintained at about 30°C, while the second compartment was maintained at a lower T_*ch*_ of about 25°C to achieve a desirable T_*ch*_ gradient. In this case, the relevant T_*ch*_ and P_*chH2O*_ are the volume-weighted averages of the two compartments.

The respiratory frequency and breathing variability, under normoxia/normocapnia, hypoxia and hypercapnia, were also analyzed. To this end, the cycle duration of each respiratory event (T_*TOT*_) was obtained by the inverse of respiratory frequency (f_*R*_; in seconds). These data were submitted to Poincaré’s analysis, where the T_*TOT*_ were plotted against the duration of the next breath.

As described by Brennan et al. (2001), the ellipse is oriented according to the line-of-identity (RR_*n*_ = RR_*n*__+__1_). To obtain the short-term variation, we calculated the width of the variation (SD1) perpendicular to line identity, using the following equation:

$${\mathrm{SD1}}^{2}=\frac{1}{2}{\text{SDSD}}^{2}$$

Where the SDSD (standard deviation of successive difference) is defined as:

$$\text{SDSD}=\sqrt{E\left[{\text{RR}}_{\text{n}}^{2}\right]-{\bar{{\text{RR}}_{\text{n}}}}^{2}}$$

The long-term variation was determined by the moving average calculated through the length (SD2) of the line identity, using:

$${\mathrm{SD2}}^{2}=2\mathrm{SDR}{\text{R}}^{2}-\frac{1}{2}{\text{SDSD}}^{2}$$

The autocovariance function is related to the variance of the RR intervals as SDRR:

$${\text{SDRR}}^{2}=\phi \mathrm{RR}\left(0\right)$$

#### Experimental Protocol: Effect of Temperature of Incubation on $\dot{V}$_*E*_, $\dot{V}$O_2_, Breathing Variability and Tb During Normocapnia, Hypoxia and Hypercapnia in Male and Female Broiler Chicks

The 3d and 14d male and female animals were placed, individually, into the respirometric chamber and allowed to move freely. For the first 30 min, during habituation, the chamber was flushed with room air (21% O_2_). After acclimation, $\dot{V}$_*E*_ and $\dot{V}$O_2_ were recorded in normocapnic/normoxic conditions for 10 min. Then, animals were exposed to a hypercapnic (7% CO_2_) or hypoxic (10% O_2_) gas mixture for 30 min. The order of mixtures was randomly chosen, with a 1-h interval of a normocapnic/normoxic mixture given between the two gaseous stimuli. The $\dot{V}$_*E*_ and $\dot{V}$O_2_ were measured at 30 min of gas exposure. During the recovery phase, the ventilatory variables were measured at 60 min. Ventilation was measured during ∼2 min after the gas exposure and $\dot{V}$O_2_ was analyzed for 2 min prior to the sealing of the animal’s chamber to record the pressure oscillations. Tb was measured before and after gas exposure.

### Determination of Body Temperature

Colonic temperature was used as a representative of Tb, and was measured by introducing a sensor (Thermistor Pod ML 309, ADInstruments^®^, Australia) into the colon. The measurements were performed at the onset and at end of the stimulus (normoxia, hypercapnia and hypoxia).

### Oxygen Consumption

The O_2_ consumption ($\dot{V}$O_2_) was measured using an open respirometry system, pull mode configuration (Mortola, 1984; Cummings et al., 2011; Patrone et al., 2018). A flowmeter (MFS; Sable Systems International, Inc.) was coupled to the plethysmography chamber outlet to control the airflow inside the chamber and to direct the outlet gas to the oxygen analyzer (ADInstruments, United States). The expired gas was dried over a column of Drierite (W.A. Hammond Drierite Co., Ltd., Xenia, OH, United States) before passing through the gas analyzer. As CO_2_ was neither analyzed nor scrubbed, oxygen consumption ($\dot{V}$O_2_) was calculated using the following equation (Koteja, 1996):

$\dot{V}$O_2_ = [FRe (FiO_2_ − FeO_2_)] / [1 − FiO_2_ (1 − RQ)]

where FRe is the end flow rate of air through the chamber, FiO_2_ is the inlet O_2_ fraction, FeO_2_ is the end O_2_ fraction, and RQ is the respiratory quotient (considered here as 0.85). The $\dot{V}$O_2_ values were presented as STPD (standard conditions of temperature, pressure and dry air).

### Determination of Monoamine Concentrations in the Brainstem and Diencephalon

After euthanasia, the brains of male and female 3d and 14d broiler chicks were rapidly excised and frozen in dry ice-cold isopentane. All samples were stored at −80°C. The brainstem and diencephalon were separated and homogenized in a solution containing 0.15 M perchloric acid, 0.1 mM ethylenediaminetetraacetic acid (EDTA), and 0.17 μM 3,4-dihydroxybenzylamine as an internal standard. The homogenates were centrifuged for 20 min at 12,000 *g* and 4°C. Protein content was determined from the pellet, and the supernatant was analyzed for NA, DA, 3,4-dihydroxyphenylacetic acid (DOPAC; the main metabolite of DA), 5-HT, and 5-hydroxyindole-3-acetic acid (5-HIAA; the main metabolite of 5-HT) by high-performance liquid chromatography coupled to an electrochemical detector (HPLC-ED), as previously described (Aquino et al., 2017; Silva et al., 2020). The chromatography separation was carried out using a C-18 column (250 mm × 4 mm, 5 μm; Merck, Darmstadt, Germany), preceded by a C18 pre-column (5 μm, 4 mm × 4 mm; Merck), and kept at 40°C. The mobile phase consisted of 100 mM NaH_2_PO_4_, 10 mM NaCl, 0.1 mM EDTA, 0.38 mM sodium 1-octanesulfonic acid, and 10% methanol in ultrapure water, pH 3.5. The pump flow rate was 1.0 mL/min, and the potential in the electrochemical detector (Decade II; Antec Scientific, Zoeterwoude, Netherlands) was set to +0.40 V vs. the Ag/AgCl reference electrode. All samples from each brain area were measured in the same analysis. The intra-assay coefficient of variation was less than 5% for all measured compounds. DA and 5-HT levels were considered to reflect neurotransmitter stock in synaptic vesicles, whereas DOPAC and 5-HIAA levels reflected neurotransmitter release (Shannon et al., 1986; Lookingland et al., 1987). DOPAC/DA and 5-HIAA/5-HT ratios were used as a measure of neurotransmitter turnover.

#### Experimental Protocols: Effect of Incubation Temperature on Heart and Lung Mass, and on Brainstem and Diencephalon Monoaminergic Concentrations, in Male and Female Broiler Chicks

Five male and five female animals (3d and 14d) from each treatment were deeply anesthetized with isoflurane and euthanized for heart, lung and brain extraction. The heart and lungs were weighed immediately, and the brain was frozen for the HPLC-ED analysis.

### Statistical Analyses

The results are reported as means ± SEM. The data were tested for normality of deviation (Cramer Von-Mises criterion) and homoscedasticity (Levene test). For monoamine data, and for body, heart and lung mass, comparisons among groups were made using one-way ANOVA. The effects of normoxia/normocapnia, hypercapnia and hypoxia on V_*T*_, f_*R*_, $\dot{V}$_*E*_, $\dot{V}$O_2_ were evaluated using two-way analysis of variance with body mass as co-variable. As $\dot{V}$_*E*_ and $\dot{V}$O_2_ change allometrically with body mass, an analysis of covariance (ANCOVA) was performed to check if the effect of the incubation temperatures was influenced by body mass of chicks in the different groups. The Tb and breathing variability, under different gases, were evaluated using two-way ANOVA. In all conditions, males and females were analyzed separately. In the case of a significant difference, means were compared using Tukey’s test. Values of *P* ≤ 0.05 were considered to be statistically significant.

## Results

### Body, Heart and Lung Mass, and Tb

Body mass (BM) of 0d, 3d, and 14d chickens is presented in Table 1. The BM of HT hatchling males was lower (*P* < 0.001) compared to CT males. 3d LT females presented a higher body mass (*P* < 0.001) compared to CT and HT chickens. In 14d LT males (*P* < 0.0001) and females (*P* = 0.005), BM was higher compared to the CT and HT groups (Table 1). In addition, 14d HT males presented a lower BM (*P* < 0.0001) compared to CT and LT males.

**TABLE 1:** Values of body temperature (Tb), heart mass (HM), lung mass (LM), and body mass (BM) of 3 and 14 days-old female and male chickens that were incubated at control (CT; 37.5°C) or at higher (HT; 39°C) or lower (LT; 36°C) temperatures during 6 h/day, from day 0 to 5 of incubation.

		LT	CT	HT	P
**Hatching**					
BM (g)	♀	51.1 ± 2.3 ^a^	50.7 ± 2.0 ^ab^	49.5 ± 3.2 ^b^	<0.005
	♂	51.7 ± 2.3 ^a^	51.3 ± 2.7 ^a^	49.3 ± 2.7 ^b^	<0.005
**3 days-old**					
Tb (°C)	♀	40.0 ± 0.2	40.0 ± 0.1	39.9 ± 0.1	ns
	♂	40.1 ± 0.2	40.1 ± 0.1	40.0 ± 0.1	ns
HM (% BM)	♀	0.6 ± 0.02	0.5 ± 0.04	0.4 ± 0.03	ns
	♂	1.0 ± 0.04	0.9 ± 0.04	1.0 ± 0.1	ns
LM (% BM)	♀	0.4 ± 0.1	0.4 ± 0.1	0.4 ± 0.01	ns
	♂	0.8 ± 0.02	0.9 ± 0.1	0.9 ± 0.1	ns
BM (g)	♀	73.7 ± 3.4 ^a^	60.2 ± 1.4 ^b^	55.5 ± 2.7 ^b^	<0.05
	♂	66.8 ± 2.9 ^a^	59.3 ± 1.8 ^ab^	57.0 ± 3.2 ^b^	<0.05
**14 days-old**					
Tb (°C)	♀	39.9 ± 0.1 ^c^	40.3 ± 0.1 ^b^	41.0 ± 0.1 ^a^	<0.05
	♂	40.1 ± 0.1 ^b^	40.3 ± 0.1 ^b^	40.8 ± 0.1 ^a^	<0.05
HM (% BM)	♀	0.8 ± 0.1 ^ab^	0.7 ± 0.1 ^b^	0.9 ± 0.1 ^a^	<0.05
	♂	0.9 ± 0.1 ^ab^	0.8 ± 0.1 ^b^	1.1 ± 0.03 ^a^	<0.05
LM (% BM)	♀	0.6 ± 0.01	0.6 ± 0.03	0.6 ± 0.03	ns
	♂	0.6 ± 0.1	0.7 ± 0.1	0.6 ± 0.02	ns
BM (g)	♀	372.6 ± 19.2 ^a^	287.6 ± 11.6 ^b^	285.7 ± 20.6 ^b^	<0.05
	♂	390.4 ± 12.3 ^a^	334.6 ± 14.1 ^b^	270.9 ± 12.9 ^c^	<0.05

*Different letters represent statistical differences in comparing chicks of the same sex and parameter. Significance level (*P* ≤ 0.05). ns, not significant (*P* > 0.05). [3d ♀ (*n* = 17); 3d ♂ (*n* = 15); 14d ♀ (*n* = 18); 14d ♂ (*n* = 17)].*

The heart and lung mass, presented in Table 1 (3d and 14d animals), are expressed as body mass percentage. No significant differences were observed among treatments for heart and lung mass of 3d animals, and lung mass of 14d male and female animals. The 14d HT male (*P* = 0.002) and female (*P* = 0.03) animals presented a higher heart mass, compared to the CT group (Table 1).

Table 1 also shows the effects of temperature of incubation on Tb under room air conditions. Thermal manipulation did not affect the Tb of 3d animals. Regarding 14d animals, incubation at HT caused an increase (*P* < 0.0001) in Tb, compared to LT and CT animals. LT females showed a lower Tb compared to other groups.

The absolute values are presented in the Supplementary Table 1.

### Ventilatory and Metabolic Variables

#### Normoxia Normocapnia

Table 2 show the ventilatory and metabolic parameters of 3d and 14d female and male chicks from the CT, LT and HT experimental groups during normoxia/normocapnia. No significant differences were observed in $\dot{V}$_*E*_, V_*T*_ or f_*R*_ among groups in females. The LT 3d males displayed a lower $\dot{V}$_*E*_ compared to CT males (*P* = 0.04), without changes in V_*T*_ and f_*R*_ (Table 2).

**TABLE 2:** Values of ventilation ($\dot{\text{V}}$_*E*_), tidal volume (V_*T*_), respiratory frequency (f_*R*_), oxygen consumption ($\dot{\text{V}}$O_2_), and ventilatory equivalent ($\dot{\text{V}}$_*E*_/$\dot{\text{V}}$O_2_) of 3 and 14 days-old female and male chickens that were incubated at control (CT; 37.5°C) or at higher (HT; 39°C) or lower (LT; 36°C) temperatures during 6 h/day, from day 0 to 5 of incubation.

		LT	CT	HT	P
**3 days-old**					
$\dot{\text{V}}$_*E*_	♀	49.6 ± 6.4	53.2 ± 4.1	57.1 ± 4.5	ns
(mL.min^–1^)	♂	38.8 ± 3.9 ^b^	54.0 ± 3.3 ^a^	47.5 ± 3.4 ^ab^	<0.05
V_*T*_	♀	0.6 ± 0.1	0.6 ± 0.1	0.6 ± 0.1	ns
(mL)	♂	0.6 ± 0.1	0.7 ± 0.1	0.6 ± 0.1	ns
f_*R*_	♀	111.5 ± 18.4	85.5 ± 11.8	91.0 ± 11.1	ns
(min^–1^)	♂	79.6 ± 13.5	105.5 ± 22.7	86.9 ± 14.0	ns
$\dot{\text{V}}$O_2_	♀	1.6 ± 0.2	1.9 ± 0.1	1.9 ± 0.1	ns
(mL.min^–1^)	♂	1.7 ± 0.2	1.7 ± 0.1	1.7 ± 0.1	ns
$\dot{\text{V}}$_*E*_/$\dot{\text{V}}$O_2_	♀	27.1 ± 2.2	29.4 ± 2.5	34.1 ± 1.8	ns
	♂	25.3 ± 2.6	32.4 ± 1.7	28.1 ± 2.4	ns
**14 days-old**					
$\dot{\text{V}}$_*E*_	♀	346.9 ± 22.5 ^a^	318.4 ± 19.3 ^a^	229.8 ± 19.9 ^b^	<0.05
(mL.min^–1^)	♂	337.8 ± 26.0	320.0 ± 18.7	249.6 ± 26.9	ns
V_*T*_	♀	5.1 ± 0.5	5.0 ± 0.4	3.4 ± 0.5	ns
(mL)	♂	5.4 ± 1.0	5.9 ± 0.5	3.2 ± 1.0	ns
f_*R*_	♀	70.5 ± 3.5	65.5 ± 5.4	70.2 ± 6.1	ns
(min^–1^)	♂	64.8 ± 6.6	57.2 ± 3.6	81.1 ± 8.1	ns
$\dot{\text{V}}$O_2_	♀	8.9 ± 0.3 ^a^	9.3 ± 0.2 ^a^	7.7 ± 0.3 ^b^	<0.05
(mL.min^–1^)	♂	10.6 ± 0.5 ^ab^	10.5 ± 0.4 ^a^	7.4 ± 0.5 ^b^	<0.05
$\dot{\text{V}}$_*E*_/$\dot{\text{V}}$O_2_	♀	38.9 ± 2.1	34.2 ± 3.1	29.6 ± 1.8	ns
	♂	33.7 ± 2.0	31.1 ± 3.1	31.1 ± 1.4	ns

*Different letters represent statistical differences in comparing chicks of the same sex and parameter. Significance level (*P* ≤ 0.05). ns, not significant (*P* > 0.05). [3d ♀ (*n* = 19); 3d ♂ (*n* = 21); 14d ♀ (*n* = 20); 14d ♂ (*n* = 20)].*

The 14d HT females presented lower $\dot{V}$_*E*_ (CT: *P* = 0.02; LT: *P* < 0.01), but no changes were observed in V_*T*_ and f_*R*_ among treatments. No significant difference were observed in the ventilatory parameters of 14d male animals, although the HT males showed a tendency to have a lower $\dot{V}$_*E*_ (*P* = 0.11) compared to other groups (Table 2).

Under normoxic/normocapnic conditions, $\dot{V}$O_2_ was not influenced by the treatments in 3d animals, but 14d HT males (CT: *P* = 0.005) and females (CT: *P* = 0.001; LT: *P* = 0.02) presented a lower metabolic rate compared to the other groups (Table 2). The ventilatory equivalent ($\dot{V}$_*E*_/$\dot{V}$O_2_) in normoxia/normocapnia was not influenced by the treatments in either sex in 3d and 14d animals.

#### Hypercapnia

**FIGURE 1:**
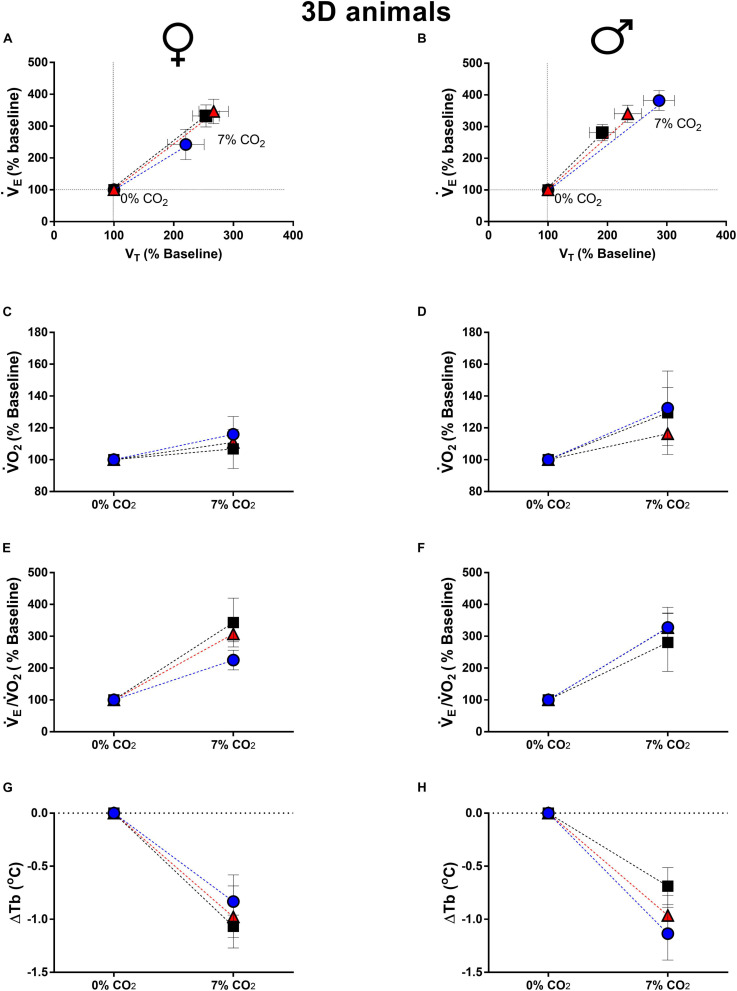
Breathing pattern **(A,B)**, oxygen consumption ($\dot{V}$O_2_) **(C,D)**, air convection requirement ($\dot{V}$_*E*_/$\dot{V}$O_2_) **(E,F)** and body temperature (Tb) **(G,H)**, during normocapnia and hypercapnia of 3-days-old female and male chickens submitted to the experimental treatments during incubation, i.e., control temperature (CT, 37.5°C), low temperature (LT, 36°C, 6 h/day, from 0 to 5th day) and high temperature (HT, 39°C, 6 h/day, from 0 to 5th day). Values are expressed as mean ± SEM. Different letters represent statistical differences among the treatments. Level of significance: *P* ≤ 0.05.

**FIGURE 2:**
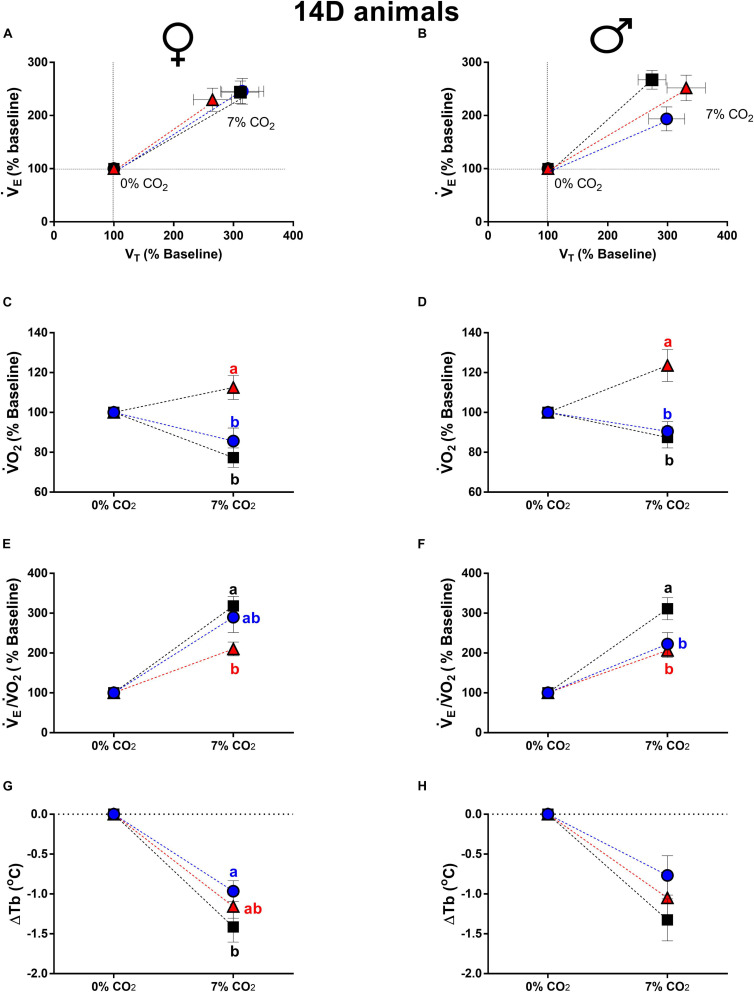
Breathing pattern **(A,B)**, oxygen consumption ($\dot{V}$O_2_) **(C,D)**, air convection requirement ($\dot{V}$_*E*_/$\dot{V}$O_2_) **(E,F)** and body temperature (Tb) **(G,H)**, during normocapnia and hypercapnia of 14-days-old female and male chickens submitted to the experimental treatments during incubation, i.e., control temperature (CT, 37.5°C), low temperature (LT, 36°C, 6 h/day, from 0 to 5th day) and high temperature (HT, 39°C, 6 h/day, from 0 to 5th day). Values are expressed as mean ± SEM. Different letters represent statistical differences among the treatments. Level of significance: *P* ≤ 0.05.

Exposure to higher levels of CO_2_ caused an increase in $\dot{V}$_*E*_, due to a higher V_*T*_, in all groups of 3d and 14d male and female animals (Figures 1, 2). None of the ventilatory parameters were affected by the treatments in 3d females, under hypercapnic conditions (Figure 1A). However, 3d LT males showed a higher V_*T*_ compared to CT (*P* = 0.04), but no significant difference was observed in $\dot{V}$_*E*_ (Figure 1B).

The thermal manipulation during incubation did not affected the metabolic rate and ventilatory equivalent of 3d females (Figures 1C,E) and males (Figures 1D,F). Hypercapnic stimulus caused a drop in Tb in both sexes, but no significant difference was observed among treatments (females: Figure 1G; males: Figure 1H).

Concerning 14d animal, the $\dot{V}$_*E*_, V_*T*_ and f_*R*_ of 14d females were not affected by the treatments (Figure 2A). On the other hand, 14d LT males showed an attenuated ventilatory response compared to CT (*P* = 0.05), with no significant differences in V_*T*_ and f_*R*_ (Figure 2B).

Differently from 3d animals, the 14d HT, females (Figure 2C) and males (Figure 2D), displayed a higher metabolic response compared to CT (females: *P* < 0.0001; males: *P* = 0.02) and LT (females: *P* = 0.0002; males: *P* < 0.0001).

The 14d HT females showed a lower $\dot{V}$_*E*_/$\dot{V}$O_2_ compared to CT (*P* < 0.003) (Figure 2E). On the other hand, 14d LT (*P* = 0.003) and HT (*P* = 0.0006) males presented an attenuated hypercapnic hyperventilation compared to CT males (Figure 2F).

Hypercapnia caused a similar decrease in Tb in all groups of 14d females and males. The only difference was observed in 14d LT females, which showed a lower drop in Tb compared to CT animals (*P* = 0.03) (Figure 2G).

#### Hypoxia

**FIGURE 3:**
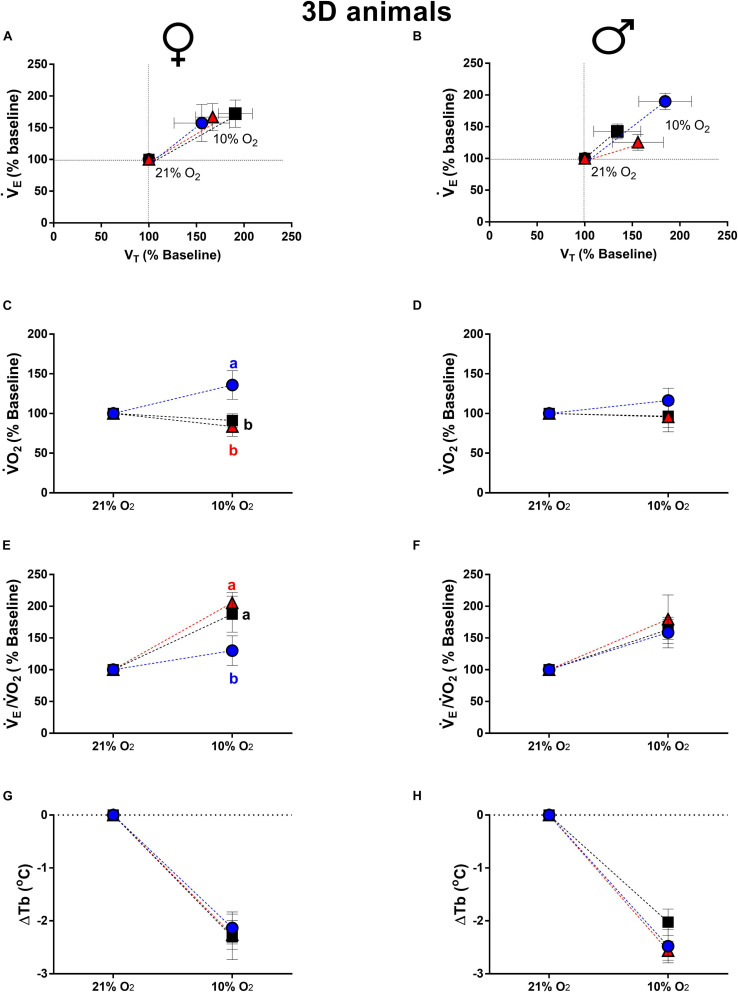
Breathing pattern **(A,B)**, oxygen consumption ($\dot{V}$O_2_) **(C,D)**, air convection requirement ($\dot{V}$_*E*_/$\dot{V}$O_2_) **(E,F)** and body temperature (Tb) **(G,H)**, during normoxia and hypoxia of 3-days-old female and male broiler chicks submitted to the experimental treatments during incubation, i.e., control temperature (CT, 37.5°C), low temperature (LT, 36°C, 6 h/day, from 0 to 5th day) and high temperature (HT, 39°C, 6 h/day, from 0 to 5th day). Values are expressed as mean ± SEM. Different letters represent statistical differences among the treatments. Level of significance: *P* ≤ 0.05.

Hypoxia increased $\dot{V}$_*E*_ in all groups of 3d females and males due to a higher V_*T*_ (Figure 3). The $\dot{V}$_*E*_ of 3d LT males was higher compared to CT (*P* = 0.02) and HT (*P* = 0.01), however, no statistical differences were observed in V_*T*_ and f_*R*_ (Figure 3B). No significant differences in the ventilatory parameters were observed among treatments under hypoxia in 3d females (Figure 3A).

The $\dot{V}$O_2_ of newly hatched chicks is presented in Figures 3C,D. The 3d LT females presented a higher $\dot{V}$O_2_ compared to CT (*P* = 0.01) and HT (*P* = 0.001) groups (Figure 3C). The $\dot{V}$_*E*_/$\dot{V}$O_2_ was also affected by low temperature of incubation, which 3d LT females showed an attenuated ventilatory equivalent compared to CT (*P* = 0.04) and HT (*P* = 0.003) groups (Figure 3E). No significant differences were observed among treatments in $\dot{V}$O_2_ and $\dot{V}$_*E*_/$\dot{V}$O_2_ of 3d males (Figures 3D,F). Hypoxia caused similar drop in Tb of 3d animals, with no statistical difference among treatments in 3d females (Figure 3G) and males (Figure 3H).

**FIGURE 4:**
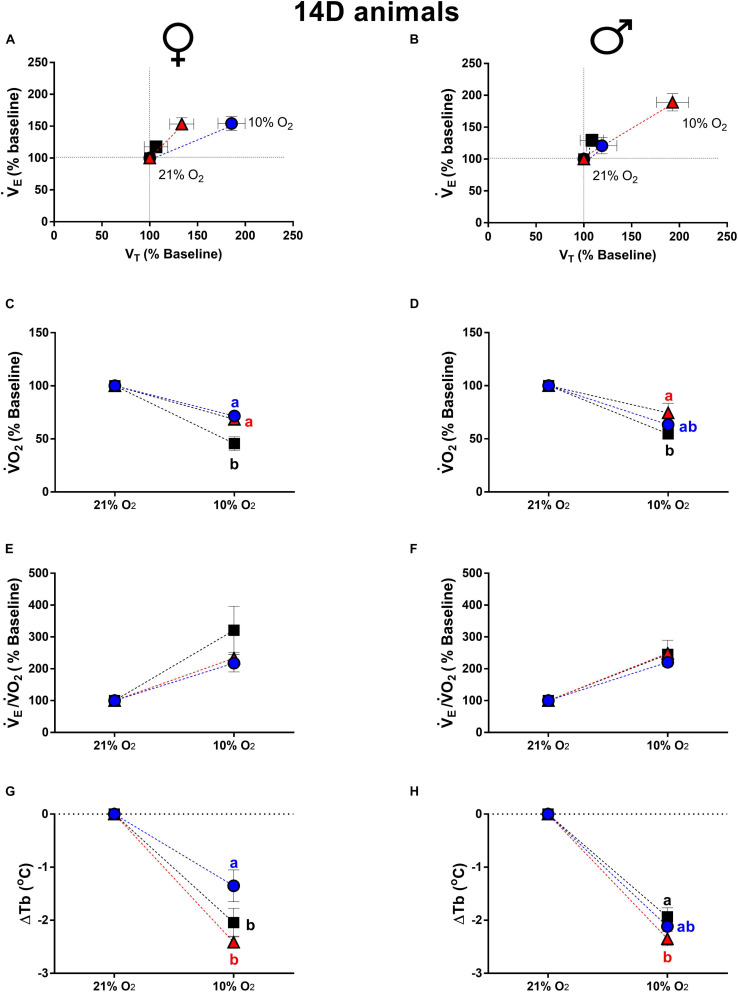
Breathing pattern **(A,B)**, oxygen consumption ($\dot{V}$O_2_) **(C,D)**, air convection requirement ($\dot{V}$_*E*_/$\dot{V}$O_2_) **(E,F)** and body temperature (Tb) **(G,H)**, during normoxia and hypoxia of 14-days-old female and male broiler chicks submitted to the experimental treatments during incubation, i.e., control temperature (CT, 37.5°C), low temperature (LT, 36°C, 6 h/day, from 0 to 5th day) and high temperature (HT, (39°C, 6 h/day, from 0 to 5th day). Values are expressed as mean ± SEM. Different letters represent statistical differences among the treatments. Level of significance: *P* ≤ 0.05.

Figure 4 shows the ventilatory, metabolic and thermal responses of 14d male and female animals during hypoxia. The 14d LT and HT females presented a higher hypoxic ventilatory response compared to CT group (LT: *P* = 0.04; HT: *P* = 0.05). The V_*T*_ of 14d LT females was higher compared to CT females (*P* = 0.004) (Figure 4A). In 14d male animals, high temperature of incubation promoted a higher ventilatory response under hypoxia compared to CT (*P* = 0.01) and LT (*P* = 0.04), modulated by a higher V_*T*_ (CT: *P* = 0.004; LT: *P* = 0.04).

Metabolic rate of 14d animals, during hypoxia, was also affected by incubation temperature. The 14d LT and HT females, displayed an attenuated drop in $\dot{V}$O_2_ compared to CT group (LT: *P* = 0.01; HT: *P* = 0.02) (Figure 4C). In 14d HT males, hypoxia caused a lower drop in $\dot{V}$O_2_ compared to CT males (*P* = 0.05) (Figure 4D).

The $\dot{V}$_*E*_/$\dot{V}$O_2_ of 14d females and males is shown in Figures 4E,F, respectively. Hypoxia increased $\dot{V}$_*E*_/$\dot{V}$O_2_ similarly in males and females and no significant difference were observed among treatments in both sexes.

Hypoxia promoted a decrease in Tb in 14d females and 14d males. The reduction in Tb in LT females was significantly smaller than the decrease measured for both the HT (*P* = 0.0003) and CT groups (*P* = 0.018) (Figure 4G). In HT males, the decrease in Tb due to hypoxia was higher compared to the CT group (*P* = 0.022) (Figure 4H).

### Breathing Variability

Regarding the breathing variability, Table 3 shows the values of SD1 and SD2 parameters used to quantify the distribution of the points under room air, hypercapnia and hypoxia, for female and male 3d and 14d chicks of each treatment (LT, CT, and HT). Under normoxia/normocapnia, no significant differences were observed among the treatments for any of the parameters evaluated, except for 14d LT (*P* = 0.0121) and HT (*P* = 0.0034) male animals, which presented a lower SD1 compared to control animals, and for 3d LT female animals, which showed a lower SD2 (*P* = 0.026) compared to HT group.

**TABLE 3:** The variability of breath duration (mean ± SEM) during room air (21% O_2_), hypercapnia (7% CO_2_), and hypoxia (10% O_2_) exposure of 3- and 14-days-old male and female chicks incubated under control (CT; 37.5°C) or at higher (HT; 39°C) or lower (LT; 36°C) during 6 h/day, from day 0 to 5 of incubation. Standard deviation (SD1 and SD2).

		Room air			7% CO_2_			10%O_2_		
Age		LT	CT	HT	LT	CT	HT	LT	CT	HT
3d	♀									
	SD1	60.1 ± 9.8	60.1 ± 8.0	72.5 ± 9.1	31.0 ± 4.0*	33.9 ± 2.0*	29.5 ± 2.8*	41.6 ± 7.1^b^	67.4 ± 6.3^a^	48.5 ± 3.5^ab^
	SD2	67.6 ± 13.5^b^	62.9 ± 8.8^ab^	81.2 ± 12.0^a^	28.6 ± 4.7*	37.0 ± 2.5	39.9 ± 5.9*	44.3 ± 9.3^b^	59.8 ± 4.3^ab^	58.3 ± 6.0^a^
	♂									
	SD1	111.2 ± 25.9	67.9 ± 23.8	61.4 ± 7.2	29.4 ± 6.3*	29.0 ± 4.4	24.9 ± 2.6	54.6 ± 5.2	53.1 ± 4.4	60.8 ± 10.3
	SD2	121.5 ± 26.6	72.3 ± 24.8	76.8 ± 10.9	36.7 ± 7.1*	31.9 ± 6.4	28.0 ± 2.4	60.6 ± 5.7	53.5 ± 5.1	71.1 ± 11.4
14d	♀									
	SD1	50.8 ± 5.6	55.1 ± 10.2	46.1 ± 8.1	77.5 ± 9.0	54.3 ± 11.0	57.7 ± 13.2	46.0 ± 8.9	44.3 ± 7.1	37.1 ± 8.4
	SD2	72.7 ± 4.2	74.6 ± 12.0	67.6 ± 13.3	112.0 ± 17.3	84.9 ± 21.6	66.5 ± 11.4	64.9 ± 12.2	60.6 ± 14.3	34.6 ± 8.1*
	♂									
	SD1	32.8 ± 6.2 ^b^	68.4 ± 6.7 ^a^	29.6 ± 3.1 ^b^	80.8 ± 11.3 ^a^*	59.7 ± 9.9^a^	32.1 ± 6.8^b^	35.6 ± 9.2	56.1 ± 12.1	25.3 ± 2.2
	SD2	44.4 ± 7.3	83.7 ± 9.2	41.1 ± 4.9	117.9 ± 26.2 ^a^*	91.6 ± 21.1^ab^	44.9 ± 10.2^b^	47.0 ± 13.8	66.5 ± 16.7	32.8 ± 2.4

Different letters in the lines represent statistical differences of responses to a gas mixture among LT, CT, and HT groups. * Indicates statistical differences of each treatment pre and post gas mixture exposure. Significance level (*P* ≤ 0.05). [3d ♀ (*n* = 19); 3d ♂ (*n* = 21); 14d ♀ (*n* = 20); 14d ♂ (*n* = 20)].

Under 7% CO_2_, 14d LT (*P* = 0.003) and CT (P = 0.041) male animals presented a higher SD1, and 14d LT male animals presented a higher SD2, compared to the HT group (*P* = 0.020; Table 3). No significant differences were observed among treatments in the 3d male and female animals or the 14d female animals. During hypoxia, the only difference was observed in 3d LT female animals, which showed a lower SD1 (*P* = 0.035) when compared to the control group, and a lower SD2 parameter (*P* = 0.026) when compared to the 3d HT female animals (Table 3).

Although thermal manipulation during incubation had a slight effect on breathing variability, high CO_2_ and low O_2_ concentrations affected this parameter in both sexes and ages. During hypercapnia, 3d LT (*P* = 0.014), CT (*P* = 0.029) and HT (*P* = 0.0003) female animals presented a lower SD1 compared to room air conditions. The SD1 value of 3d LT males was also reduced by the hypercapnic mixture (*P* < 0.05); however, 14d LT males presented a higher SD1 compared to the same treatment under normocapnia (Table 3). The SD2 parameter was also affected by 7% CO_2_ exposure in both ages and sexes. The 3d LT (*P* = 0.011) and HT (*P* = 0.007) females and 3d LT (*P* = 0.001) males presented a lower SD2 compared to room air, while 14d LT male animals presented a higher SD2 (*P* = 0.029) under the same conditions.

Unlike with hypercapnia, the only group that was affected by hypoxia was the 14d HT females, which had a smaller SD2 (*P* = 0.0085) compared to normoxia (Table 3).

**FIGURE 5:**
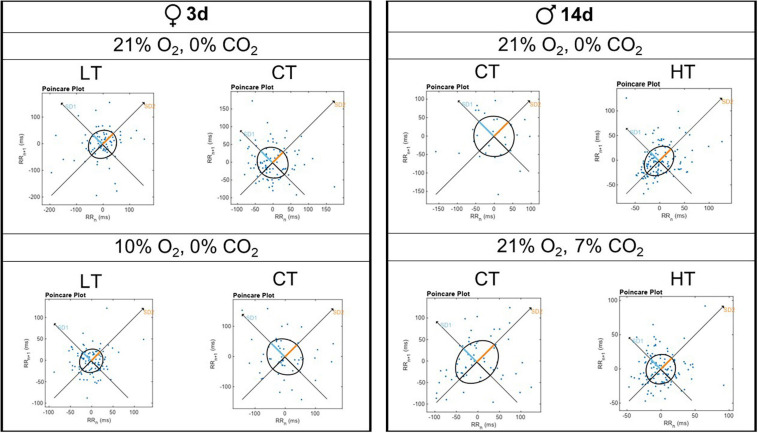
Representative Poincaré plot graphs for breathing variability during normoxia/normocapnia (21% O_2_/0% CO_2_), hypercapnia (7% CO_2_) and hypoxia (10% O_2_) of a 3d female and a 14d male.

Figure 5 shows representative Poincaré plots of breathing variability for 3d LT and CT females under room air and hypoxia; and 14d HT and CT males under room air and hypercapnia. Under hypoxic/normocapnic situation, 3d LT females showed a lower breathing variability comparing to controls (left side). On the right side, 14d HT males displayed a lower breathing variability under both situations (normocapnic/normoxic and hypercapnic/normoxic conditions).

### Brain and Diencephalon Monoaminergic Concentrations

Tables 4, 5 show the levels of NA, DA, DOPAC, DOPAC/DA ratio, 5-HT, 5-HTIAA, and 5-HTIAA/5-HT ratio in the brainstem and diencephalon, respectively. The only differences observed were in brainstem DOPAC and the DOPAC/DA ratio of 3d animals (Table 3). Thermal manipulations (both CT and HT) promoted an increase (*P* = 0.006) in the DOPAC/DA ratio in 3d females, whereas HT 3d males displayed an increase (*P* = 0.039) in the brainstem DOPAC levels, compared to LT animals (Table 4).

**TABLE 4:** Effects of low (LT), control (CT), and high (HT) temperature of incubation on noradrenaline (NA), dopamine (DA), 3,4-dihydroxyphenylacetic acid (DOPAC), DOPAC/DA, serotonin (5-HT), 5-hydroxyindole-3-acetic acid (5-HIAA), and 5-HTIAA/5-HT in the brainstem of 3- and 14-days-old female and male chicken.

		LT	CT	HT	P
**3 days-old**					
NA (pg/μg)	♀	5.7 ± 0.8	6.1 ± 0.5	5.4 ± 0.4	ns
	♂	5.5 ± 0.7	4.9 ± 0.8	5.2 ± 0.6	ns
DA (pg/μg)	♀	0.4 ± 0.07	0.4 ± 0.08	0.3 ± 0.03	ns
	♂	0.3 ± 0.01	0.3 ± 0.05	0.4 ± 0.06	ns
DOPAC (pg/μg)	♀	0.3 ± 0.04	0.2 ± 0.04	0.2 ± 0.03	ns
	♂	0.1 ± 0.02 ^a^	0.2 ± 0.02 ^ab^	0.2 ± 0.04 ^b^	<0.05
DOPAC/DA	♀	0.7 ± 0.06 ^a^	0.5 ± 0.1 ^b^	0.7 ± 0.03 ^*a*^	<0.001
	♂	0.5 ± 0.05	0.6 ± 0.08	0.4 ± 0.05	ns
5-HT (pg/μg)	♀	2.2 ± 0.3	2.2 ± 0.1	2.2 ± 0.2	ns
	♂	2.5 ± 0.2	2.0 ± 4.5	2.2 ± 0.2	ns
5-HTIAA (pg/μg)	♀	1.1 ± 0.1	1.0 ± 0.09	1.4 ± 0.1	ns
	♂	1.4 ± 0.2	1.1 ± 0.2	1.2 ± 0.2	ns
5-HTIAA/5-HT	♀	0.5 ± 0.05	0.4 ± 0.04	0.6 ± 0.05	ns
	♂	0.5 ± 0.04	0.7 ± 0.2	0.5 ± 0.08	ns
**14 days-old**					
NA (pg/μg)	♀	11.4 ± 2.6	8.5 ± 1.0	8.9 ± 1.4	ns
	♂	7.5 ± 0.9	6.7 ± 0.9	7.8 ± 0.8	ns
DA (pg/μg)	♀	1.0 ± 0.2	0.8 ± 0.3	0.7 ± 0.1	ns
	♂	0.6 ± 0.03	0.5 ± 0.05	0.8 ± 0.1	ns
DOPAC (pg/μg)	♀	0.3 ± 0.03	0.4 ± 0.1	0.2 ± 0.03	ns
	♂	0.2 ± 0.02	0.2 ± 0.04	0.3 ± 0.06	ns
DOPAC/DA	♀	0.3 ± 0.03	0.4 ± 0.04	0.4 ± 0.09	ns
	♂	0.4 ± 0.05	0.4 ± 0.06	0.3 ± 0.04	ns
5-HT (pg/μg)	♀	8.5 ± 1.5	6.6 ± 2.0	5.2 ± 0.6	ns
	♂	4.9 ± 0.4	5.3 ± 0.4	4.4 ± 0.6	ns
5-HTIAA (pg/μg)	♀	1.6 ± 0.4	1.4 ± 0.2	0.8 ± 0.1	ns
	♂	1.1 ± 0.05	1.2 ± 0.1	1.0 ± 0.1	ns
5-HTIAA/5-HT	♀	0.2 ± 0.03	0.2 ± 0.03	0.2 ± 0.04	ns
	♂	0.2 ± 0.02	0.2 ± 0.02	0.2 ± 0.02	ns

*Different letters represent statistical differences in comparing chicks of the same sex and parameter. Significance level (*P* ≤ 0.05). ns, not significant (*P* > 0.05). [3d ♀ (*n* = 17); 3d ♂(*n* = 15); 14d ♀ (*n* = 18); 14d ♂ (*n* = 17)].*

**TABLE 5:** Effects of low (LT), control (CT), and high (HT) temperature of incubation on noradrenaline (NA), dopamine (DA), 3,4-dihydroxyphenylacetic acid (DOPAC), DOPAC/DA, serotonin (5-HT), 5-hydroxyindole-3-acetic acid (5-HIAA), and 5-HTIAA/5-HT in the diencephalon of 3- and 14-days-old female and male chicken.

		LT	CT	HT	P
**3 days-old**					
NA (pg/μg)	♀	3.1 ± 0.3	3.6 ± 0.5	3.0 ± 0.5	ns
	♂	3.3 ± 0.4	3.3 ± 0.3	2.9 ± 0.4	ns
DA (pg/μg)	♀	0.4 ± 0.05	0.6 ± 0.1	0.5 ± 0.09	ns
	♂	0.3 ± 0.01	0.6 ± 0.03	0.7 ± 0.3	ns
DOPAC (pg/μg)	♀	0.2 ± 0.02	0.2 ± 0.03	0.2 ± 0.03	ns
	♂	0.2 ± 0.02	0.2 ± 0.02	0.2 ± 0.04	ns
DOPAC/DA	♀	0.4 ± 0.02	0.4 ± 0.07	0.6 ± 0.08	ns
	♂	0.4 ± 0.04	0.3 ± 0.03	0.4 ± 0.05	ns
5-HT (pg/μg)	♀	3.7 ± 0.4	5.2 ± 1.2	3.6 ± 0.5	ns
	♂	4.3 ± 0.6	4.2 ± 0.2	4.0 ± 0.3	ns
5-HTIAA (pg/μg)	♀	1.1 ± 0.1	1.1 ± 0.2	1.3 ± 0.1	ns
	♂	1.3 ± 0.1	1.2 ± 0.1	1.2 ± 0.2	ns
5-HTIAA/5-HT	♀	0.3 ± 0.04	0.2 ± 0.05	0.4 ± 0.02	ns
	♂	0.3 ± 0.04	0.3 ± 0.05	0.3 ± 0.04	ns
**14 days-old**					
NA (pg/μg)	♀	5.9 ± 0.7	6.2 ± 0.6	5.8 ± 0.9	ns
	♂	5.5 ± 0.3	5.4 ± 0.5	4.3 ± 1.0	ns
DA (pg/μg)	♀	1.1 ± 0.2	1.0 ± 0.09	0.9 ± 0.2	ns
	♂	1.2 ± 0.2	1.0 ± 0.05	0.8 ± 0.2	ns
DOPAC (pg/μg)	♀	0.2 ± 0.03	0.2 ± 0.03	0.2 ± 0.02	ns
	♂	0.3 ± 0.04	0.3 ± 0.02	0.3 ± 0.04	ns
DOPAC/DA	♀	0.3 ± 0.01	0.2 ± 0.03	0.2 ± 0.03	ns
	♂	0.2 ± 0.02	0.3 ± 0.01	0.3 ± 0.07	ns
5-HT (pg/μg)	♀	6.8 ± 0.8	7.3 ± 0.8	6.4 ± 0.8	ns
	♂	7.8 ± 0.8	7.6 ± 0.5	5.4 ± 1.3	ns
5-HTIAA (pg/μg)	♀	0.8 ± 0.1	0.9 ± 0.1	0.6 ± 0.03	ns
	♂	0.9 ± 0.09	0.8 ± 0.06	0.7 ± 0.1	ns
5-HTIAA/5-HT	♀	0.1 ± 0.01	0.1 ± 0.02	0.1 ± 0.01	ns
	♂	0.1 ± 0.01	0.1 ± 0.003	0.1 ± 0.02	ns

*Significance level (*P* ≤ 0.05). ns, not significant (*P* > 0.05). [3d ♀ (*n* = 17); 3d ♂ (*n* = 15); 14d ♀ (*n* = 18); 14d ♂ (*n* = 17)].*

## Discussion

In the present study, we evaluated the effects of thermal manipulation during the first 5 days of embryonic development on ventilation, metabolism and thermal responses to hypercapnia and hypoxia in 3- and 14-day-old chickens (males and females), as well as on brain monoamine concentrations. We demonstrated that incubation at a high temperature during a “critical window” of organogenesis (Burggren, 1998) decreases $\dot{V}$_*E*_ (females), decreases $\dot{V}$O_2_ (females and males), and increases Tb in 14d chickens under room air conditions, practically regardless of sex. In younger animals, females are more affected by thermal manipulation than males. Furthermore, both hot and cold incubation increased brainstem DA turnover in 3d females, but not in males.

### Body, Lung and Heart Mass

The 14d HT animals, both males and females, presented a higher heart mass compared to CT animals, suggesting that a greater effort was required to pump blood to the tissues, possibly due to an inefficiency in gas exchange, since $\dot{V}$_*E*_ was decreased in females (significant) and males (tendency). Although no effect of the treatments was observed on lung mass, environmental challenges during embryonic development have been shown to interfere with lung morphology. For instance, do Amaral-Silva et al. (2019) described that chicks incubated under hypoxia between day 12 and day 18 of incubation presented a morphological parabrochial remodeling, characterized by increased volume density and respiratory surface area of structures involved in gas exchange. In addition, broilers incubated under higher temperatures from E7–E21 presented a greater mortality by ascites (Molenaar et al., 2011). In fact, this metabolic disorder is closely associated with pulmonary hypertension and right ventricle hypertrophy (Molenaar et al., 2011).

Cold incubation increased BM in 3d females, and in both males and females at 14 days. Chicken embryos can compensate their growth later on after periods of growth delay caused by cold (Mortola, 2009). Likewise, Nyuiadzi et al. (2020) observed that males exposed to a lower incubation temperature presented a higher body mass under cold exposure during rearing, while females incubated in a cold temperature have greater growth at normal post-hatching temperatures. These effects of cold incubation on BM are possibly a result of hormonal differences between sexes in the very early embryo development and in the early growth phases (Dainat et al., 1991; Kocamis et al., 1998; Bello et al., 2014; Nyuiadzi et al., 2020). A reduction in body weight was observed in 3d HT males, with no compensation exhibited during the subsequent 14-day growth period. Nevertheless, it was not clear why males were more negatively affected by hot incubation than females. Perhaps different sex hormone profiles present during early phases of incubation, as was shown recently by Wang et al. (2019), might be the cause of this sex difference.

### Brain Monoamines

Numerous stressors have been shown to be associated with changes in brain monoamine metabolism. A previous study showed that acute heat and cold exposure of turkeys increases central NA turnover (El Halawani et al., 1973). More recently, Hamasu et al. (2012) demonstrated that restraint with isolation-induced stress stimulates only brain dopaminergic metabolism in 1-day-old male layer chicks. Here, we observed that cold and hot incubation increases brainstem DA activity in 3d female chickens, confirming that younger females are more vulnerable to central thermal manipulation than younger males. No changes were observed in NA or 5-HT. Similarly, both lower and higher incubation temperatures during days 0–14 were found to cause an increase in plasmatic DA in embryo chicks (von Blumröder and Tönhardt, 2002).

### Breathing Variability

Thermal manipulation alone (LT and HT) decreased breathing variability in 14d males under normoxic/normocapnic conditions, by reducing the short-term respiratory variability, with no changes in SD2. In the other groups, thermal manipulation did not evoke changes in breathing variability (in both sexes at 3d, and in females at 14d). These data suggest that changes in incubation temperature during early developmental stages may cause alterations in the brainstem respiratory network, responsible for respiratory rhythm modulation. In fact, the chick embryonic hindbrain starts to generate rhythmic activities at E4 (Fortin et al., 1995), which means that the development and maturation of this network may have been affected by our treatments, promoting the changes in cycle duration. The 14d HT males also presented a decrease in breathing variability during 7% CO_2_ by reducing both short- and long-term respiratory variability. On the other hand, 3d LT females subjected to hypoxia presented lower short-term breathing variability compared to control female animals of the same age. These results resemble a previous study, in which newborn rats exposed to hypoxia and cold presented a decrease in inter-breath variability (Cameron et al., 2000).

Our data suggest that changes in this parameter are sex- and age-dependent, as female animals subjected to thermal manipulation presented changes in variability a few days after hatching, while male animals showed these alterations in cycle duration belatedly. Other studies from our lab also observed differences in breathing variability between 7- and 8-day-old male and female rats subjected to brainstem CA neuron lesions, with males showing a decrease in breathing variability, unlike with females (Patrone et al., 2018). Interestingly, female animals exposed to a cold temperature during incubation showed a reduced breathing variability only just after hatching, possibly due to the maturation of medullary respiratory network, since no significant differences were observed in these parameters in 14d female chicks.

### Pulmonary Ventilation and Metabolism

None of the treatments caused an effect on metabolic rate in 3d animals under normoxia/normocapnia. Our data corroborate the results obtained by Mortola and Toro-Velasquez (2013) from 1-day-old White Leghorn hatchlings whose eggs were incubated in cold temperatures. However, our results suggest that cold temperature during incubation promoted a lower ventilation in 3d males, but not in females at the same age. In addition, thermal manipulation does not affect the Tb of newly hatched males and females under normoxia/normocapnia, which is contrary to the result obtained by Mortola and Toro-Velasquez (2013), who found a difference in Tb and no significant difference in ventilation, in similar ambient conditions. The main difference between the two studies may be the time of thermal manipulation during embryonic development (first 5 days vs. whole incubation), but may also be related to the strains used (broilers Cobb 500 vs. White Leghorn) and the temperature reduction in the cold treatment (36^*o*^C vs. 35^*o*^C). In addition, unlike the current study, previous studies using thermal manipulation have shown that increasing the temperature of incubation decreases Tb at hatching, and for up to 70 days after hatching (Piestun et al., 2011, 2013a). However, those authors performed thermal manipulation during E7–E16.

### Chemosensitivity

Under 7% CO_2_, no significant differences were observed in ventilation, metabolism and Tb in 3d animals. However, hot incubation attenuated the hypercapnic hyperventilation due to an increase in $\dot{V}$O_2_ in 14d females and males, with no change or a small effect in $\dot{V}$_*E*_. In fact, these animals presented a higher heart mass compared to control animals, suggesting a higher effort to pump blood to the tissues that have a higher metabolic rate. Interestingly, juvenile chickens (21–23 days old) exposed to the elevated incubation temperature during early development (38.6°C—between incubation day 0 and 5) challenged with a heat stress presented an increased corticosterone release, suggesting that early heat exposure modifies the organization of the hypothalamic–pituitary–adrenal (HPA) axis (Wilsterman et al., 2015).

Regarding the 14d LT animals, males also have an attenuation of CO_2_-hyperventilation; however, by using a different strategy. These animals have a lower increase in $\dot{V}{}_{E}$, with no change in $\dot{V}O{}_{2}$, suggesting a reduction in respiratory chemosensitivity. This change in chemosensitivity is likely due to alteration in central areas, since no change in hypoxic ventilatory response was observed in cold incubated 14d males. To detect changes in CO_2_/pH, birds possess peripheral (located in the carotid bodies), central (located in the central nervous system), and intrapulmonary chemoreceptors that are highly sensitive to CO_2_ (Milsom, 2002).

The organogenesis of main organs, like the brain and heart, occurs very early during incubation, and changes in temperature during this critical phase may impact normal development and maturation of the respiratory control system, including the CO_2_ sensitivity (Szdzuy and Mortola, 2008; Burggren and Reyna, 2011). As far as we know, there are no studies showing the evolution of maturation of neural ventilation mechanisms in birds. On the other hand, in neonatal rats the percentage of neurons stimulated by elevated CO_2_ was significantly greater in animals older than 12 days, compared to younger animals (Wang and Richerson, 1999; Putnam et al., 2005). A previous study performed in our lab (Espinha et al., 2014), also observed an age-dependent hypercapnic ventilatory response in female broiler chicks, suggesting that some mechanisms, involved in the ventilatory response to CO_2_, may change during post-hatch life.

Interestingly, although the 14d HT animals have a higher metabolic rate under hypercapnia, no significant difference was observed in Tb comparing to the controls. In addition, 14d LT females showed a lower decrease in Tb under hypercapnia, despite no changes in $\dot{V}$O_2_ were observed. This effect in Tb may be caused by changes in thermoregulation mechanisms. In the context, Morita et al. (2016) observed that chicks incubated under low temperature displayed a decrease in vascularity in neck, back and thighs skin, which can hinder the heat loss.

Hypoxia induced hyperventilation in all groups. Concerning newly hatched animals, the only difference was observed in 3d LT females, which presented an attenuated hyperventilatory response due to a lower reduction in $\dot{V}$O_2_, compared to CT and HT animals. Therefore, it seems that for newly hatched LT females to increase $\dot{V}$_*E*_ or to maintain Tb is more energetically costly. Even with the higher $\dot{V}$O_2_ observed in LT animals during hypoxia, the Tb decreased with the same magnitude of the other groups, which indicates that cold incubation might have decreased heat conservation/increased heat loss, in comparison to CT animals at this age. Since the hypoxic ventilatory response was similar among LT and CT females, these changes in $\dot{V}$O_2_ should not depend on the carotid bodies, but possibly on a central mechanism. These results are in agreement with available data in turkey, chicken and Muscovy duck hatchlings subjected to low temperatures during the last days of incubation, which all showed increased heat production compared to controls (Minne and Decuypere, 1984; Tzschentke and Nichelmann, 1999; Nichelmann et al., 2001). Other studies involving environmental alterations during chicken embryonic development, also observed an attenuated hyperventilatory response during hypoxia. However, different from our results, the authors observed that the changes in hypoxic hyperventilation was due to a blunted ventilation and not by $\dot{V}$O_2_ alteration, suggesting that hypoxia during the whole or in the last third of incubation can interfere in normal development of peripheral chemoreceptors (Szdzuy and Mortola, 2007; Ferner and Mortola, 2009). Therefore, environmental conditions during embryonic development can cause a temporal variation in the developmental sequence of regulatory mechanisms that will depend on the critical window that the stimulus was applied and also the type of stimulation.

Unlike 3d LT females, the same treatment induced 14d females to present a greater decrease in Tb under hypoxic conditions compared to other groups. In addition, the hypoxic-hypometabolic response was attenuated in 14d LT females, indicating that they had a lower metabolic reaction to hypoxia, which seems to be disadvantageous for survival in those conditions (Mortola and Dotta, 1992; Mortola, 2004; Lu et al., 2005). Reductions in Tb should facilitate metabolic depression during hypoxia by reducing temperature-dependent O_2_ demands (Scott et al., 2008). These lower depressive effects of hypoxia on Tb and metabolism in thermally manipulated chickens may be necessary for maintaining the high ventilatory response to low-O_2_ conditions. In fact, these animals presented a higher hypoxic ventilatory response (significant in females, and a tendency in males), which may have contributed to the attenuation of the hypometabolic response.

The 14d HT females and males, also displayed an attenuation in hypoxic-hypometabolic response compared to CT animals, which reflected in an increase in ventilation to supply a higher oxygen demand. Curiously, despite the lower drop in $\dot{V}$O_2_, the hot-incubated animals showed an acute drop in Tb, once again suggesting that the thermoregulatory mechanisms may be affected by the temperature of incubation.

## Conclusion

In summary, as far as we know, our results, for the first time, point to a phenotypic plasticity in ventilation, breathing variability, metabolic rate, Tb and brainstem DA activity in newly hatched and juvenile broiler chicks subjected to different incubation temperature. Thermal manipulation affects more the hyperventilation induced by hypercapnia than the hypoxic challenge induced by changing ventilation, which may suggest an effect on central or intrapulmonary chemosensitivity. In general, females are more affected by thermal manipulation than males, indicating that a sexual dimorphism is already present at this age.

## Data Availability Statement

The raw data supporting the conclusions of this article will be made available by the authors, without undue reservation.

## Ethics Statement

The animal study was reviewed and approved by the protocols were performed according to CONCEA (“Conselho Nacional de Controle de Experimentação Animal”; National Council for Animal Care Control) and approved by the local animal care committee (CEUA—Comissão de Ética no Uso de Animais—FCAV-UNESP; Protocol: 011955/18).

## Author Contributions

AR, LHG, MM, RS, and KB contributed to interpretation, drafted, and revised critically for important intellectual content. CC-S also contributed to drafted and revised critically for important intellectual content. AR, CC-S, CT, KC, and VL contributed to acquisition and analysed of data. AR and LHG contributed to conception and designed of the work. All authors contributed to the article and approved the submitted version.

## Conflict of Interest

The authors declare that the research was conducted in the absence of any commercial or financial relationships that could be construed as a potential conflict of interest.
