# Supplementary material for: Embryonic Thermal Manipulation Affects Ventilation, Metabolism, Thermal Control and Central Dopamine in Newly Hatched and Juvenile Chicks
Source: Front Physiol. 2021 Jun 17;12:699142. doi: 10.3389/fphys.2021.699142 (PMC8249324; doi:10.3389/fphys.2021.699142)
Supplement: Supplementary file 1 [file Table_1.DOCX]

**Supplementary material**

| Table 1. Values of heart mass (HM), lung mass (LM) and body mass (BM) of 3 and 14 days-old female and male chickens that were incubated at control (CT; 37.5ºC) or at higher (HT; 39ºC) or lower (LT; 36ºC) temperatures during 6 hours/ day, from day 0 to 5 of incubation. | | | | | | | |
| --- | --- | --- | --- | --- | --- | --- | --- |
|  |  |  |  | **LT** | **CT** | **HT** | P |
| **3 day-old** | | |  |  |  |  |  |
|  | **HM (g)** | | ♀ | 0.6 ± 0.01 ^a^ | 0.5 ± 0.01 ^b^ | 0.5 ± 0.01 ^b^ | < 0.05 |
|  |  |  | ♂ | 0.5 ± 0.02 | 0.5 ± 0.02 | 0.5 ± 0.02 | ns |
|  | **LM (g)** | | ♀ | 0.4 ± 0.03 | 0.4 ± 0.03 | 0.4 ± 0.03 | ns |
|  |  |  | ♂ | 0.4 ± 0.02 | 0.5 ± 0.02 | 0.5 ± 0.02 | ns |
|  | **BM (g)** | | ♀ | 73.7 ± 3.4 ^a^ | 60.2 ± 1.4 ^b^ | 55.5 ± 2.7 ^b^ | < 0.05 |
|  |  |  | ♂ | 66.8 ± 2.9 ^a^ | 59.3 ± 1.8 ^ab^ | 57.0 ± 3.2 ^b^ | < 0.05 |
|  |  |  |  |  |  |  |  |
| **14 day-old** | | |  |  |  |  |  |
|  | **HM (g)** | | ♀ | 2.5 ± 0.1 | 2.1 ± 0.1 | 2.4 ± 0.1 | ns |
|  |  |  | ♂ | 2.7 ± 0.1 ^a^ | 2.2 ± 0.1 ^b^ | 2.8 ± 0.1 ^a^ | < 0.05 |
|  | **LM (g)** | | ♀ | 1.7 ± 0.1 | 1.6 ± 0.1 | 1.7 ± 0.1 | ns |
|  |  |  | ♂ | 1.8 ± 0.1 | 2.0 ± 0.1 | 1.8 ± 0.1 | ns |
|  | **BM (g)** | | ♀ | 372.6 ± 19.2 ^a^ | 287.6 ± 11.6 ^b^ | 285.7 ± 20.6 ^b^ | < 0.05 |
|  |  |  | ♂ | 390.4 ± 12.3 ^a^ | 334.6 ± 14.1 ^b^ | 270.9 ± 12.9 ^c^ | < 0.05 |
| Different letters represent statistical differences in comparing chicks of the same sex and parameter. Significance level (P ≤ 0.05). ns, not significant (P > 0.05). [3d ♀ (n=17); 3d ♂ (n=15); 14d ♀ (n=18); 14d ♂ (n=17)]. | | | | | | | |
